# Compact organic liquid dielectric resonator antenna for air pressure sensing using soft material

**DOI:** 10.1038/s41598-020-72021-7

**Published:** 2020-09-10

**Authors:** Jen-Hahn Low, Pei-Song Chee, Eng-Hock Lim, Kim-Yee Lee

**Affiliations:** 1 Department of Electrical and Electronic Engineering, Universiti Tunku Abdul Rahman, 43000 Kajang, Malaysia; 2 Department of Mechatronics and Biomedical Engineering, Universiti Tunku Abdul Rahman, 43000 Kajang, Malaysia

**Keywords:** Electrical and electronic engineering, Polymers, Fluids

## Abstract

For the first time, a flexible and deformable liquid dielectric resonator antenna (LDRA) is proposed for air pressure sensing. The proposed LDRA can be made very compact as it has employed liquidized organic dielectric with high dielectric constant (~ 33) with low loss tangent (~ 0.05). Here, a soft elastomer container has been fabricated using soft-lithography method for holding the liquid, and an air cavity is tactfully embedded into the central part of a cylindrical DRA to form an annular structure that can be used for sensing air pressure. It will be shown that the inclusion of the air cavity is essential for making the antenna structure sensitive to pressure changes. Simulations and experiments have been conducted to verify the functionalities of the proposed organic LDRA as microwave radiator and as air pressure sensor. It has been proven to have higher antenna gain than the water LDRA in the frequency range of 1.8–2.8 GHz, while achieving a good air pressure sensitivity of 270 MHz/bar.

## Introduction

Pressure sensor is a critical component that has been commonly used for monitoring and controlling in areas such as biomedical technologies^1^ and auto industries^2,3^. A number of pressure sensors that operate based on different sensing mechanisms such as resistive^4^, capacitive^5^ and inductive^6^ are commercially available. These sensors, however, require the involvement of wires and batteries, which can restrict their mobility. Surface acoustic wave (SAW) pressure sensors, on the other hand, can be operated in passive mode without the need of wires and batteries by connecting them to external antennas^7,8^. However, the inclusion of the external antennas can increase the circuit areas and material costs significantly.

The rapid growth of pressure-sensing applications in harsh environments requires the use of wireless communication to transmit signals and it has generated the demand for a multifunctional antenna that can act both as a physical quantity-sensing device and as a communication device. In the past years, the microstrip patch antennas have been explored for designing as strain sensors^9,10^ and pressure sensors^11,12^. However, such metallic antennas have a higher conductor loss at high frequencies, and this has become a limiting factor. Dielectric resonator antennas (DRAs), which has no inherent conductor loss, offer higher radiation efficiency^13^ and wider bandwidth. Pure water (distilled and deionized water) was first employed as the dielectric for designing a tunable liquid DRA (LDRA) at low frequencies (~ 50 MHz)^14^. Despite its high permittivity (> 70), the loss tangent of water is too high (> 0.1) for beyond operating frequency of 1 GHz, which can be a big hurdle for designing antennas at high frequencies. Pure water also exhibits phase changes such as turning into ice if the temperature goes below 0 °C. To address these drawbacks, recently, low-loss and low freezing point organic liquids such as ethyl acetate (*ε*_*r*_ = 7.1, tan *δ* = 0.089)^15,16^ and choline l-alanine (*ε*_*r*_ ~ 8–12, tan *δ* ~ 0.02–0.1)^17^ have been employed for designing various LDRAs. Since these liquids do not have a fixed shape, they are usually contained in rigid containers made of the 3D-printed material (VeroBlue RGD840)^15,16^ or Perspex acrylic^17^. For all the cases^15–17^, the liquid dielectrics have low dielectric constants (< 20), which are not suitable to be used for designing a compact LDRA. Also, the rigid containers can limit the functionality of the antenna structure as a pressure sensor, which normally relies on the working principle of diaphragm deflection.

Stretchable materials such as polydimethylsiloxane (PDMS, Sylgard 184, Dow Corning, USA) and Ecoflex (type 0,030, Smooth-on-Inc, USA) elastomers have low stiffness and are commonly used to replace rigid container for improving the stretchability of liquid antennas^18,19^. Stretchable materials have also been utilized in other disciplines, such as micropump^20–22^, self-powered sensor^23,24^ and soft sensor^12,25,26^. With the improved stretchability, resonant frequency of a liquid antenna can now be mechanically tuned over a wide range of frequencies by deforming the elastomer and can therefore act as a wireless pressure sensor. Changes in the geometry of the embedded liquid can vary the electrical properties of the antenna, which in turn change the resonant frequency^27,28^.

For the first time, a dual-functional LDRA that has high dielectric constant is proposed for sensing air pressure. As compared to our previous research involving liquid patch antenna for sensing air pressure^12^, in this project, organic acetonitrile (*ε*_*r*_ ~ 32.8 and tan *δ* ~ 0.05325) with low loss tangent is employed for designing a LDRA at 2.4 GHz. High compactness is achievable because of the use of high-permittivity liquid dielectric. To our best knowledge, this is the first demonstration of the application of high-permittivity and low-loss liquid dielectric for designing a LDRA. To contain the acetonitrile, a stretchable and deformable elastomer Ecoflex container is constructed using soft-lithography method. For performing air-sensing functionality, an air cavity is tactfully embedded into a cylindrical DRA to form an annular structure. It will be shown that the air cavity is essential to make the proposed antenna structure sensitive for responding to changes in air pressure. The air cavity expands at low pressure, changing the shape of the LDRA, and it causes its resonant frequency to increase. This feature will be employed for sensing the air pressure effectively. Different from the previously mentioned sensors which are only able to sense air pressure, we have proposed a novel structure that combines the antenna and pressure sensing functionalities into a single piece. This multifunctional module is compact in circuit footprint and low in material costs. On top of that, we have constructed the module using the soft-lithography technique and it has the advantage of rapid prototyping. We have also numerically proven the integration of an air cavity into the dual-functional antenna structure can increase its pressure sensing sensitivity significantly.

## Methods

### Dielectric characterization and material selection

**Figure 1:**
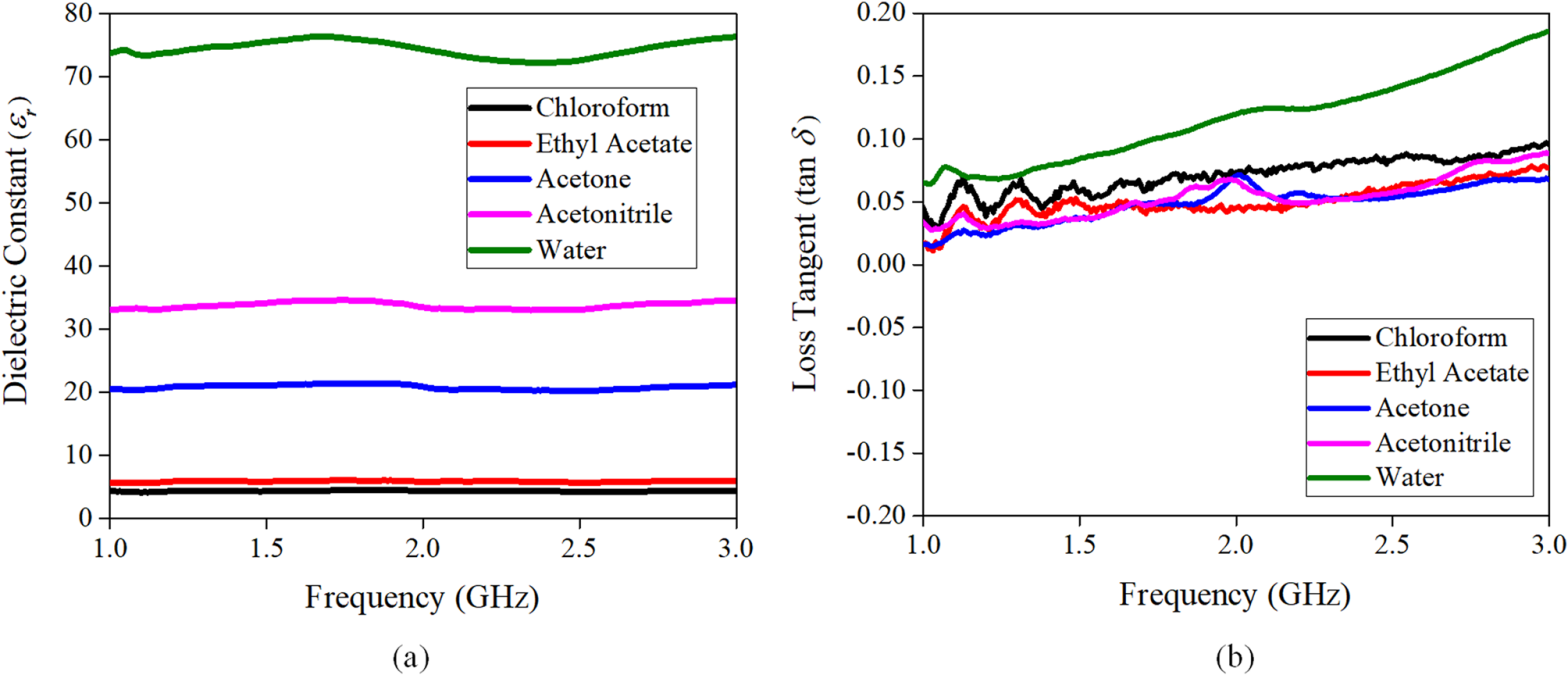
Measured (**a**) dielectric constants and (**b**) loss tangents for the five liquid samples.

It is essential to know the dielectric characteristics of the liquids before employing them for designing a LDRA. Different types of liquids such as chloroform, ethyl acetate, acetone, acetonitrile, and deionized water are characterized in the frequency range of 1–3 GHz. An open-ended coaxial probe^29^ has been employed for measuring dielectric constant and loss tangent. The measured dielectric constants and loss tangents are shown in Fig. 1a,b, respectively. It can be seen that water has the highest dielectric constant of 72.25 at 2.4 GHz; however, it cannot be used for designing an antenna due to its high loss tangent of ~ 0.13. The dielectric constants of chloroform (*ε*_*r*_ ~ 4.32, tan *δ* ~ 0.082) and ethyl acetate (*ε*_*r*_ ~ 5.77, tan *δ* ~ 0.056) are a bit too low for designing a compact LDRA. With reference to Fig. 1, it can be seen that acetone (*ε*_*r*_ ~ 20.28, tan *δ* ~ 0.052) and acetonitrile (*ε*_*r*_ ~ 32.8, tan *δ* ~ 0.053) have reasonably high dielectric constants and low loss tangents that can be used for designing a compact LDRA to achieve good antenna performances. Since acetonitrile has a higher dielectric constant, it is chosen for designing a compact LDRA for this project. Furthermore, the freezing point of acetonitrile is – 45 °C, where this characteristic provides a possibility for the LDRA to work at cold climate areas without the need of adding antifreeze, which is needed for water antenna^30^.

### Fabrication processes

**Figure 2:**
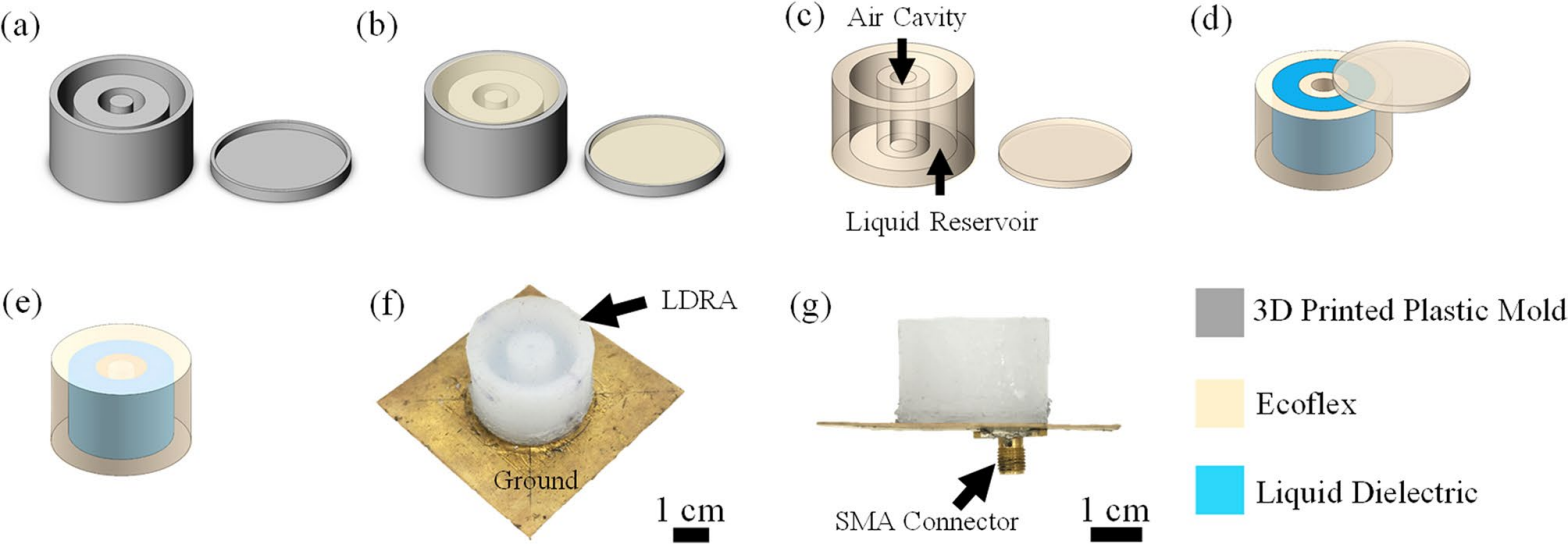
The fabricating processes of the LDRA. (**a**) 3D printed plastic molds. (**b**) Pouring Ecoflex solution into the molds. (**c**) Removing Ecoflex container from the molds. (**d**) Pouring liquid dielectric (acetonitrile) into the Ecoflex container. (**e**) Sealing up the container. (**f**) The LDRA is placed on a copper-based ground plane and soldered with an SMA connector. (**g**) Side view of the completed LDRA.

Figure 2 shows the fabrication processes of the proposed LDRA. It is started with the fabrication of the flexible container for holding the liquid dielectric, which is made using the soft-lithography technique. First, two plastic molds were printed with acrylonitrile butadiene styrene (ABS) material using a 3D printer (Ultimaker 3, Netherlands), as shown in Fig. 2a. This printer has a stated layer resolution of 20–200 μm (with a 400 μm nozzle), with XYZ resolutions of 12.5 μm, 12.5 μm, and 2.5 μm, respectively. The printed molds have good surface uniformity and they are able to produce good replicas^31,32^. Instead of applying the conventional PDMS (Sylgard 184), which has been commonly employed for fluidic antennas, we are using Ecoflex 0030 (Smooth-on-Inc, USA) (elastic modulus, *E* = 125 kPa) for making the flexible container. Such material enables a larger degree of stretchability as it has higher elasticity than the PDMS with a mixing ratio of 10:1 (base to crosslinker) (elastic modulus, *E* = 750 kPa). Here, Ecoflex solution with a mixing ratio 1:1 (resin to hardener) was poured into the molds (see Fig. 2b) and degassed in a desiccator for 10 min to remove trapped air bubbles. Trapped air bubbles in the Ecoflex solution can degrade the mechanical strength of the container significantly. Later, they were left alone for 4 h at room temperature to cure.

Figure 2c depicts the cured Ecoflex container and cover lid, which were carefully disassembled from the plastic molds. The cured container has a liquid reservoir and an air cavity. Cured Ecoflex 0030 has low dielectric constant (2.02) and low loss tangent (0.0508). Then, the liquid reservoir was filled up with acetonitrile (dielectric constant of *ε*_*r*_ = 32.8 at 2.4 GHz, Sigma-Aldrich, USA) to form the LDRA, as shown in Fig. 2d, and the air cavity was filled up with ambient air. The air cavity expands when the external air pressure is decreased, and vice versa, and this feature will be used for sensing changes in air pressure. Finally, as can be seen in Fig. 2e, the container was sealed with an Ecoflex-made cover lid using silicon adhesive (Sil-poxy, Smooth-on-Inc, USA). Sealing process is important as it can prevent liquid from leaking and evaporating away. The acetonitrile is volatile at room temperature and it has a boiling point of 82 °C. In this design, nevertheless, the liquid dielectric is stored in an enclosed cavity and vaporization is negligible. The container, which is made of the soft Ecoflex 0030 silicone elastomer, is also able to offer good chemical^33^ and impact resistances^34^. Finally, a copper-made ground plane (60 mm × 60 mm) was attached beneath the LDRA using silicon double sided adhesive (Adhesives Research Inc., USA), which was followed by the soldering of a SMA connector to enable the probe feeding (Fig. 2g). The connector was then connected to a vector network analyzer (VNA) for further measurements.

## Antenna configuration and working principle

**Figure 3:**
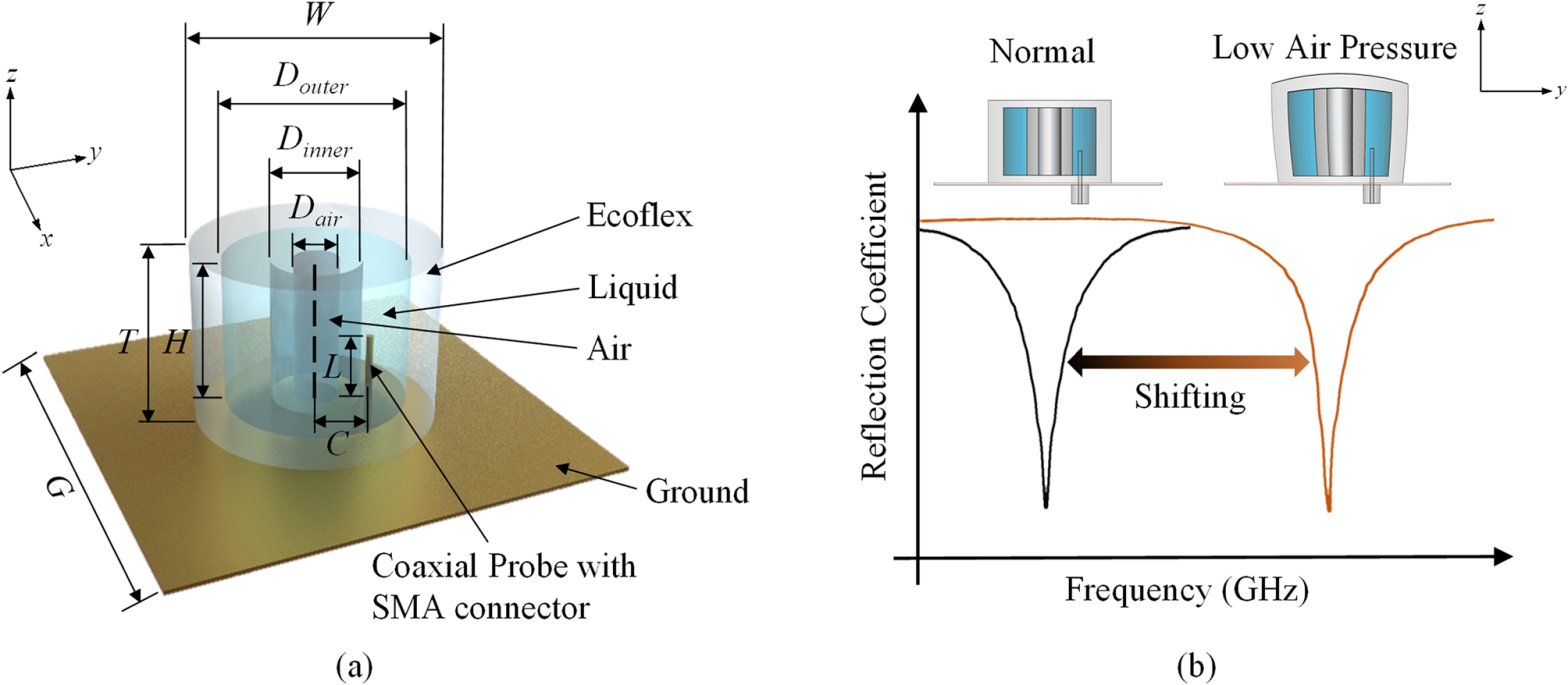
(**a**) 3D view of the proposed LDRA. (**b**) The effect of changing external air pressure on its resonant frequency.

The 3D view of the proposed annular-shaped organic LDRA, as shown in Fig. 3a, is fed by a coaxial probe at an offset distance of *C* = 8 mm from the center. The LDRA has a diameter of *W* = 32.4 mm and a height of *T* = 21.9 mm, with a square copper plate with length of *G* = 60 mm employed as ground plane. The operating principle of the proposed antenna structure is briefly described here. It is able to function as an air pressure sensor to detect low pressure condition (< 0 bar gauge pressure), as can be seen in Fig. 3b. When the ambient pressure reduces, air that is contained in the air cavity expands and it deforms the liquid reservoir’s structure, resulting in a change in the antenna volume, and it shifts the resonant frequency of the LDRA.

**Figure 4:**
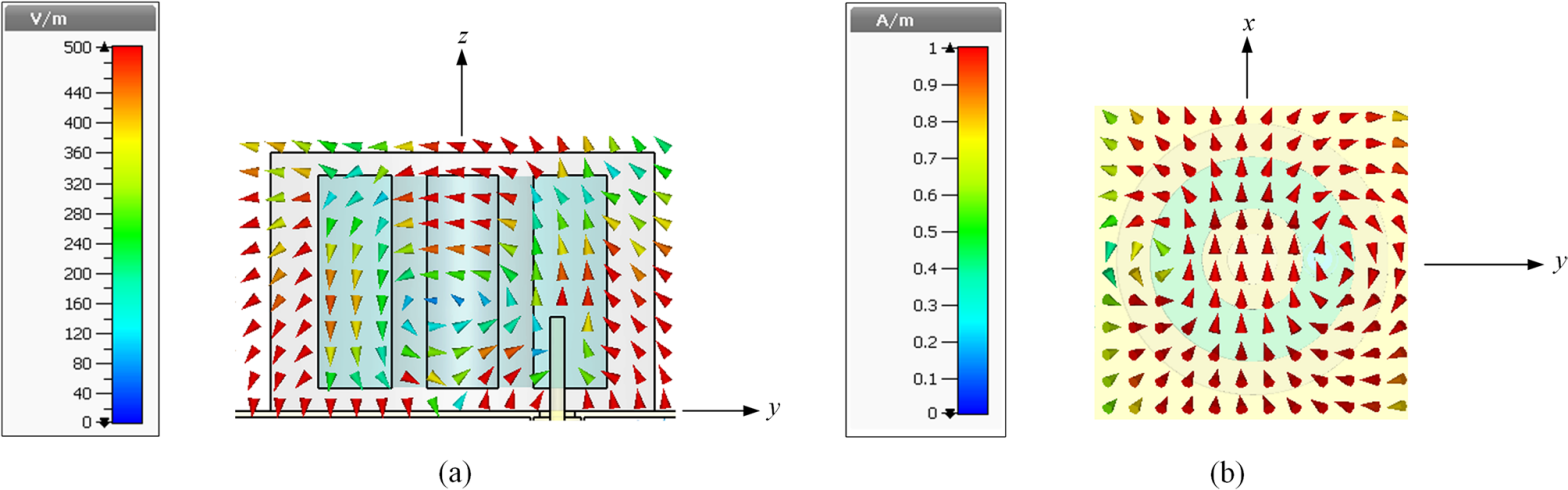
Distributions of the simulated (**a**) electric fields and (**b**) magnetic fields of the LDRA at 2.4 GHz.

All of the simulations are done using the CST Microwave Studio. The design procedure starts from a cylindrical DRA with a diameter of *D*_*outer*_ and a height of *H*, which is mounted on a ground plane, and the resonant frequency (*f*) of its HEM_11δ_ mode^35^ can be calculated by (1).

1 $$f= \frac{6.321c}{\pi {D}_{outer}\sqrt{{\varepsilon }_{r}+2}}\left[0.27+0.36\left(\frac{{D}_{outer}}{4H}\right)+0.02{\left(\frac{{D}_{outer}}{4H}\right)}^{2}\right]$$

where *c* is the speed of light and *ε*_*r*_ is the dielectric constant of the DRA. By using (1), the HEM_11δ_ mode of a cylindrical DRA with diameter of *D*_*outer*_ = 24.4 mm, height of *H* = 17.9 mm, and *ε*_*r*_ = 32.8 is calculated to be 1.656 GHz (simulation: 1.616 GHz). In fact, the annular DRA is also able to be calculated using (1) for its HEM_11δ_ mode^36^. Since the central portion is removed from the dielectric resonator, the actual resonant frequency of an annular DRA is usually higher than that of the cylindrical DRA. In our design, the liquid part of the LDRA is intended to be designed in an annular shape with a height of *H* = 17.9 mm, outer diameter of *D*_*outer*_ = 24.4 mm, inner diameter of *D*_*inner*_ = 12 mm, and a relative dielectric constant of *ε*_*r*_ = 32.8. Without including the Ecoflex, the simulated frequency of the liquid part is found to be 1.868 GHz. Then, the Ecoflex soft material is used to construct a container for holding the liquid, shifting the resonant frequency to 2.398 GHz. After removing the central portion of the Ecoflex to form the air cavity (*D*_*air*_ = 6 mm), the frequency will then shift to 2.402 GHz due to the decrease in dimension. It will be shown that the air cavity at the center can be used for air pressure sensing, which will be discussed in the later part. Finally, the impedance matching of the LDRA can be further improved by adjusting the length of the probe (*L* = 8 mm). As can be justified from the simulated resonant electric and magnetic field distributions shown in Fig. 4a,b, respectively, the proposed LDRA is excited in its fundamental HEM_11δ_ mode^37,38^.

## Results

### Radiation characterization

**Figure 5:**
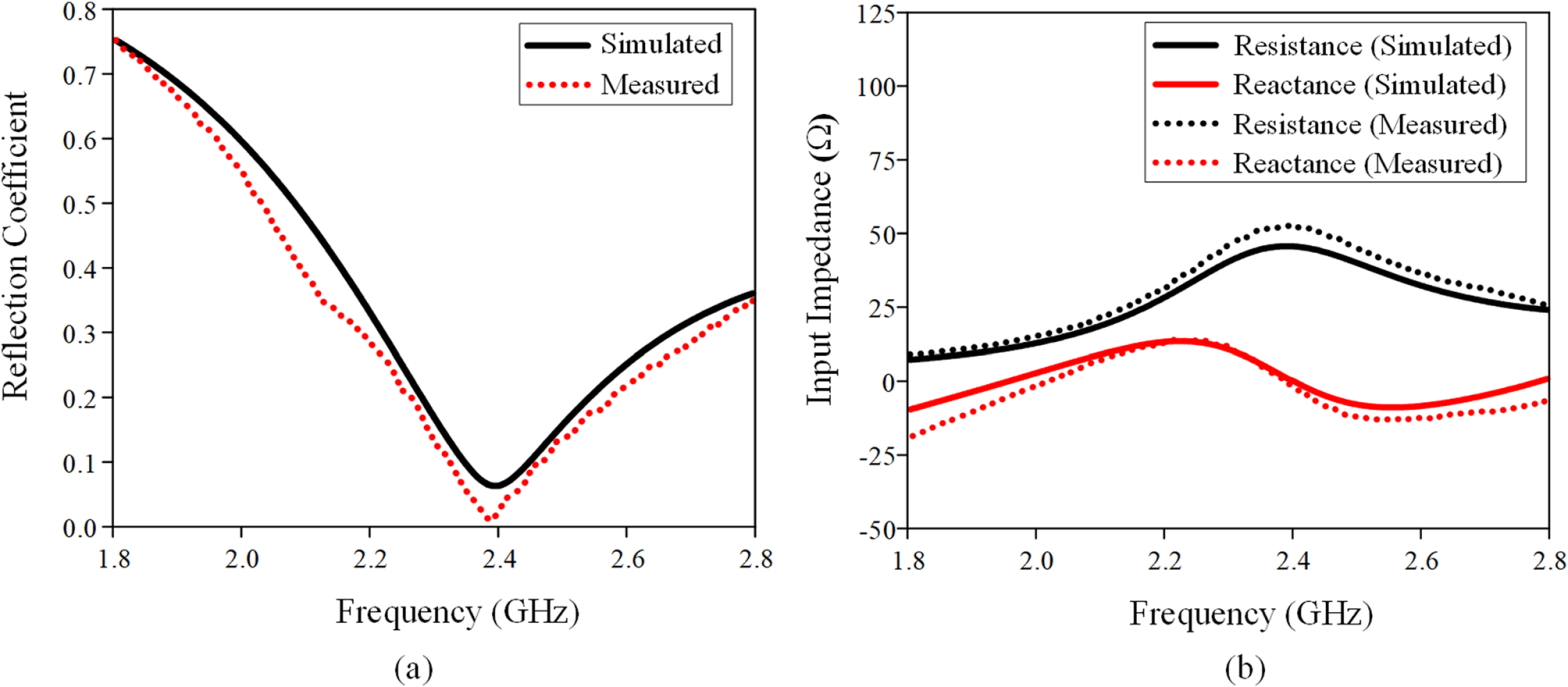
Measured and simulated (**a**) reflection coefficients and (**b**) input impedances of the LDRA.

**Figure 6:**
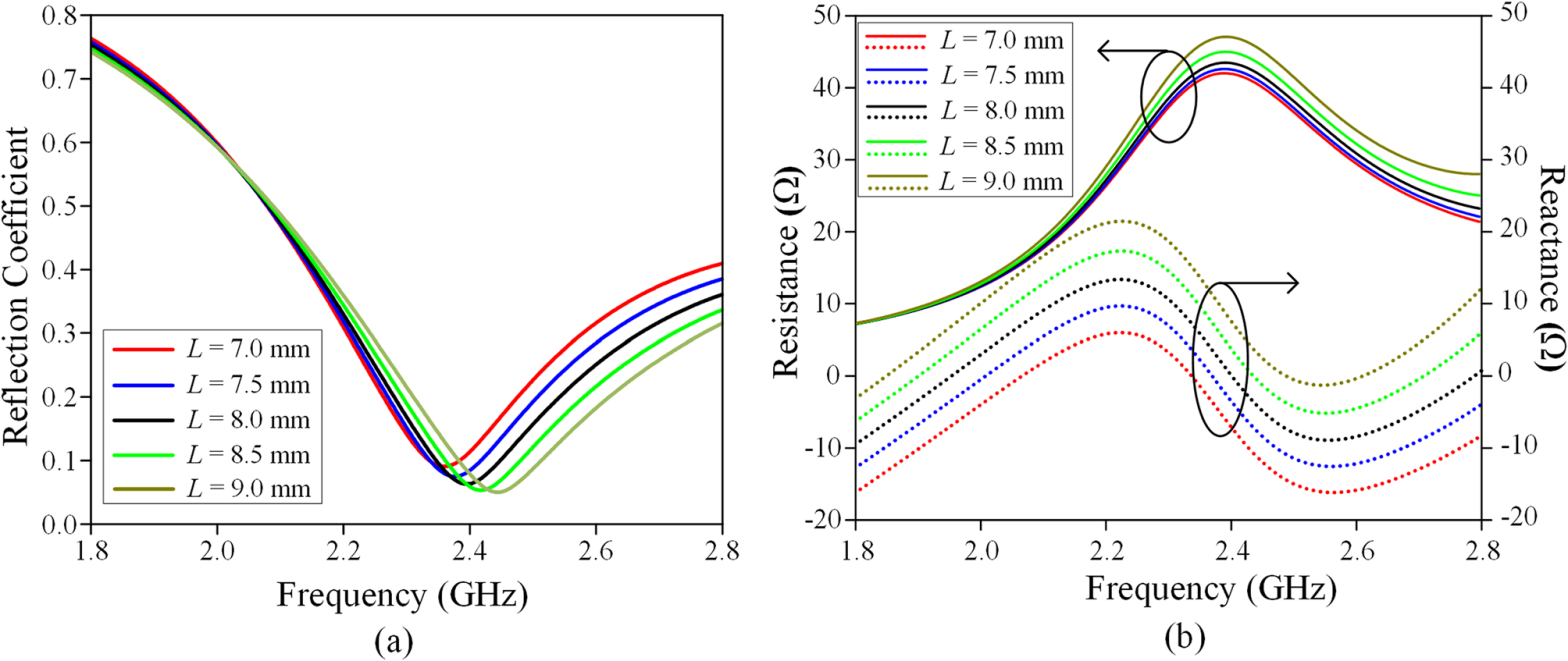
Simulated effects of varying probe length (*L*) on the (**a**) reflection coefficient and (**b**) input impedance.

**Figure 7:**
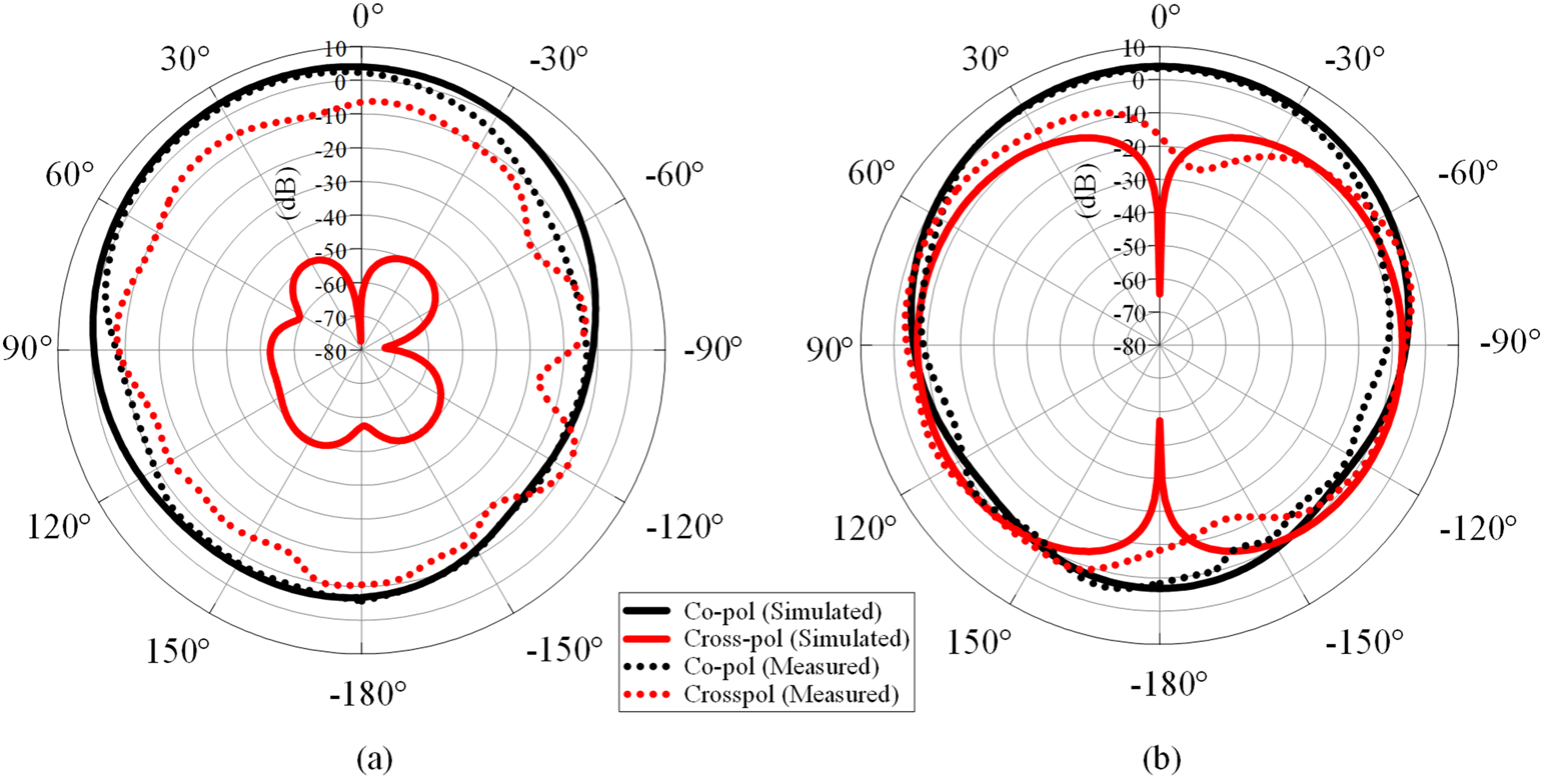
Measured and simulated radiation patterns of the LDRA in the (**a**) *E*-plane and (**b**) *H*-plane at 2.4 GHz.

The measured and simulated reflection coefficients and input impedances are shown in Fig. 5a,b respectively, showing reasonable agreement. The dominant HEM_11δ_ mode is found to be at 2.4 GHz, covering a measured bandwidth of 24.3% (simulated bandwidth: 20.4%). The measured and simulated Q factors are found to be 4.12 and 4.90, respectively. Similar range (3.64 to 10.2) of Q factor is also found in other antennas^39–41^. With reference to Fig. 5a, the measured reflection coefficient shows a better matching level than the simulated one because the measured resistance is much closer to 50 Ω (shown in Fig. 5b). Actually, the difference between the simulated and measured input impedances is quite small. The minor discrepancy may be due to fabrication tolerances. A parametric analysis for different probe lengths (*L*) has been conducted to identify the optimum value. Here, *L* is varied from 7.0 to 9.0 mm, with the corresponding reflection coefficients and input impedances shown in Fig. 6a,b, respectively. In this case, the resonant frequency shifts from 2.36  to 2.44 GHz, showing that increasing probe length causes the resonant frequency to increase, with a better matching. Better matching level is achievable for the longer probe, as can be seen in Fig. 6b, due to the increment of the antenna resistance from 41 to 45 Ω, making it closer to the port impedance of 50 Ω. However, it also causes the resonant frequency to deviate from 2.4 GHz. In our design, *L* = 8 mm is chosen as the optimum probe length as it can produce a resonant frequency close to 2.4 GHz, with sufficiently good matching level. The measured and simulated radiation patterns are shown in Fig. 7 at the resonant frequency of 2.4 GHz. Broadside radiation patterns are observed, as expected for this mode. High cross-polarization is observed in the *H*-plane (Fig. 7b) due to the probe-fed mechanism that is employed by this design. Similar effect is also seen in other research papers^42,43^. According to the explanation provided in reference^43^, vertical electric fields introduced by the feeding probe can contribute to the increase of cross-polarization.

**Figure 8:**
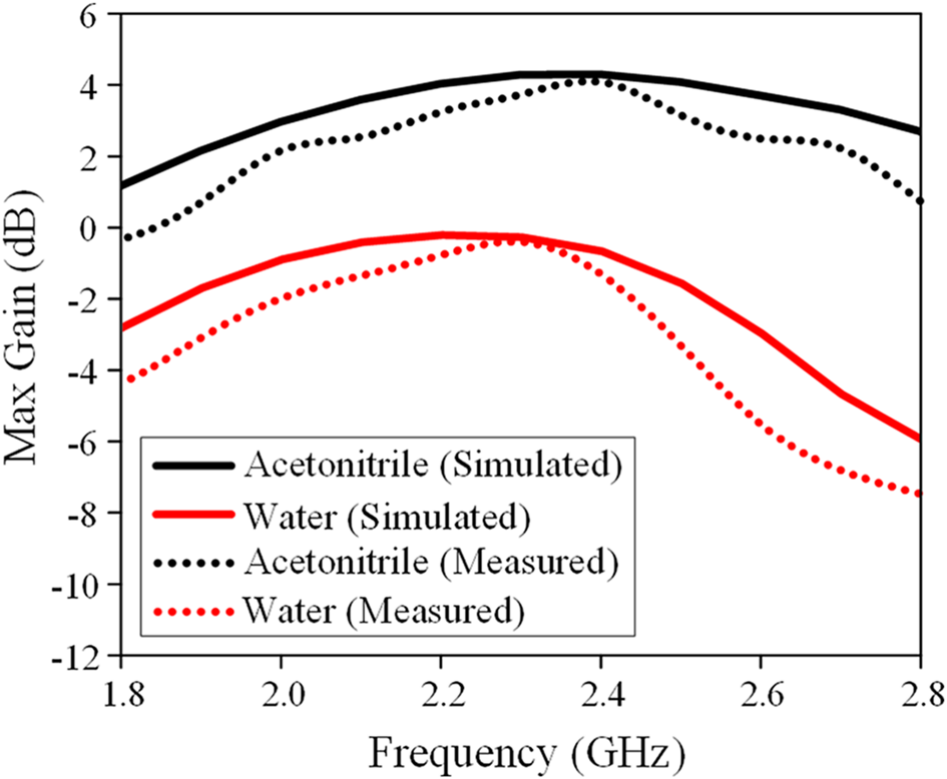
Measured and simulated antenna gains of the acetonitrile and water LDRAs.

A similar LDRA is also constructed using deionized water in the same frequency range for comparison purpose. The antenna gains of the water and acetonitrile LDRAs are compared in Fig. 8. In general, the acetonitrile LDRA has higher antenna gain (~ 2 dB) than the water LDRA. This is because acetonitrile has lower loss than water in this frequency range.

Antenna efficiencies (HEM_11δ_ mode) of the proposed LDRA are simulated for the cases with and without including mismatch loss. To have the same antenna volume, all of the dimensions are fixed and only the liquid dielectric is replaced. The results are provided in Table 1, along with other important information such as dielectric constant, loss tangent, and resonant frequency. It can be seen that the antenna efficiency is inversely proportional with the loss tangent. Water has the highest loss tangent (0.130), as a result, it has the poorest antenna efficiency. Most others are able to go beyond 70%. The lower the loss tangent of a liquid dielectric, the higher the antenna efficiency is. With reference to the table, the total antenna efficiency (including impedance mismatch loss) of the proposed LDRA, which is made of acetonitrile, is 72.04% at 2.4 GHz. Although the resonant frequency of the HEM_11δ_ mode shifts when a different liquid dielectric is used for designing the LDRA, its radiation characteristics such as electric and magnetic field distributions as well as the corresponding radiation patterns are maintaining the same.

**Table 1:** Comparison of LDRAs made of different dielectric liquids (all are made with the same dimension).

Dielectric liquid	Dielectric constant, *ε*_*r*_	Loss tangent, tan *δ*	Resonant frequency of HEM_11δ_ (GHz)	Radiation efficiency excluding impedance mismatch loss (%)	Total efficiency including impedance mismatch loss (%)
Water	72.25	0.130	1.71	29.37	29.02
Acetonitrile	32.80	0.053	2.40	72.33	72.04
Acetone	20.28	0.052	2.89	74.08	74.06
Ethyl acetate	5.77	0.056	4.42	72.11	72.06
Chloroform	4.32	0.082	4.72	70.78	68.94

### Pressure sensing

**Figure 9:**
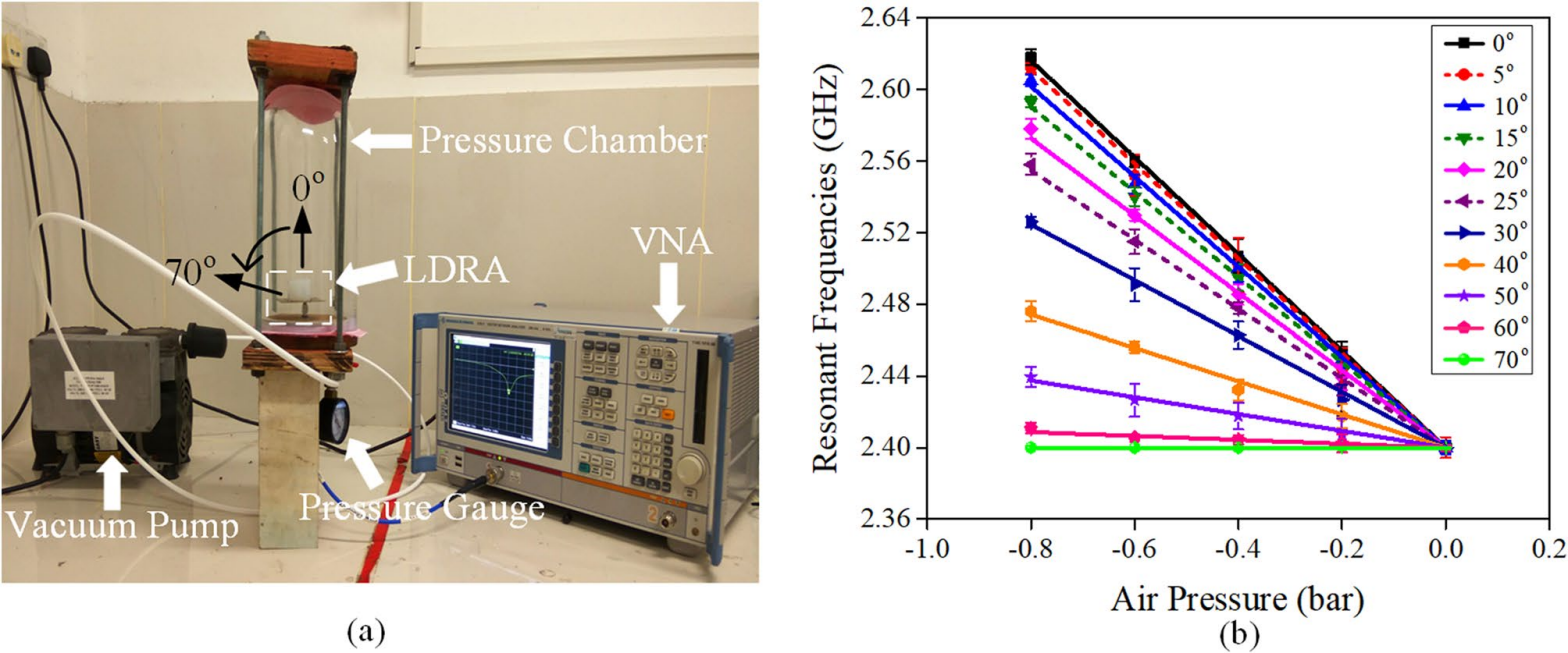
(**a**) Experimental setup for sensing changes in air pressure. (**b**) Resonant frequencies of the LDRA as a function of air pressure change at different placement angles.

Experimental setup for changing the ambient pressure is shown in Fig. 9a, where a vacuum pump and a pressure gauge are engaged for controlling the air pressure in a pressure chamber. The pressure in context refers to the gauge pressure which is zero-referenced against ambient pressure. Figure 9b shows that the resonant frequency of the antenna increases linearly when the ambient pressure is reduced. Different placement angles have been studied and a comparison is shown in Fig. 9b. Placement angle of 0° shows the highest sensitivity (270 MHz/bar). The sensitivity decreases as the tilting angle is increased until it is reaching zero response at 70° (Supplementary Table S1). This is because the liquid in the reservoir can produce different forces on the walls of the air cavity at a different placement angle due to the influence of gravity. This feature can be employed for sensing the ambient pressure surrounding the LDRA. The effect of the altitude (level above the sea level) of the sensor is minimal, according to (2), where *P* is the atmospheric pressure at a given altitude of *h*^44^. This effect can be eliminated through calibration when a new measurement is performed. Ambient factor such like temperature can also affect the pressure sensing mechanism, but it can be easily removed through proper calibration. The air pressure responses will be simulated using multi-physical simulation software and analyzed shortly. Due to the limitation of our pressure chamber, experiment on increasing air pressure cannot be done.

2 $$P \left(bar\right)=1.01325{(1-2.25577\times {10}^{-5}\times h)}^{5.25588}$$

## Discussion

**Figure 10:**
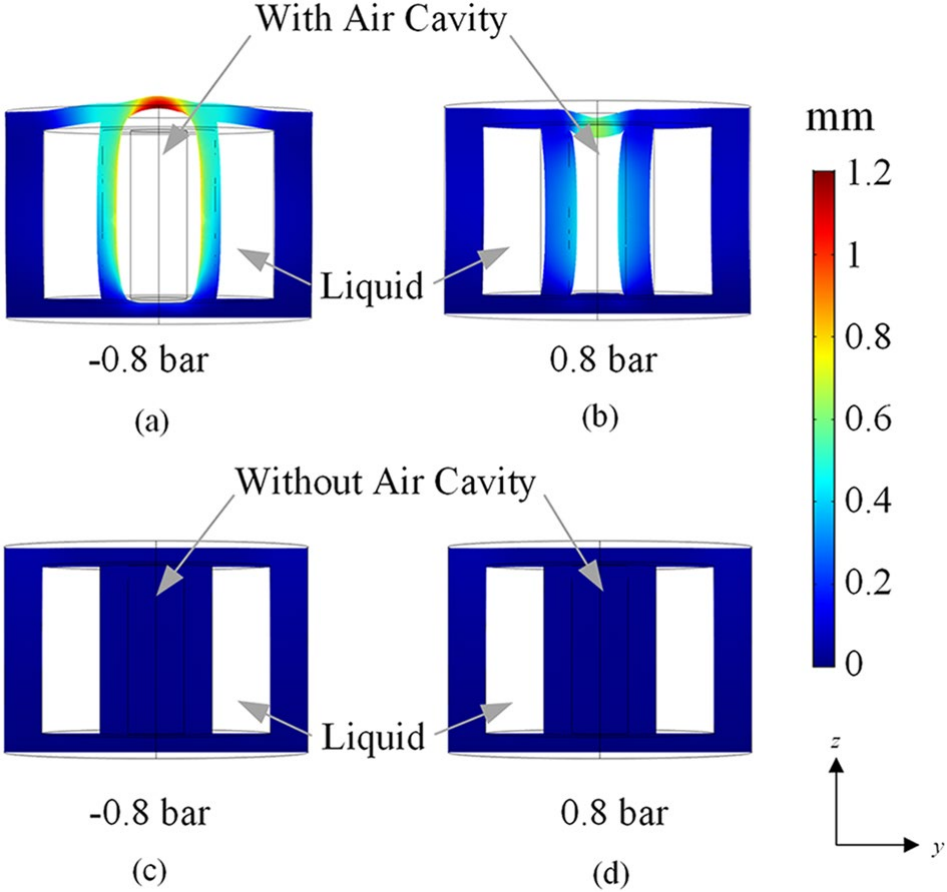
Simulated shape deformation of the acetonitrile LDRA with an air cavity under a surrounding ambient pressure of (**a**) − 0.8 bar and (**b**) 0.8 bar; Simulated shape deformation of the acetonitrile LDRA without an air cavity under a surrounding ambient pressure of (**c**) − 0.8 bar and (**d**) 0.8 bar.

Shape deformation of the proposed acetonitrile LDRA is also simulated and analyzed using the COMSOL Multiphysics software. It is studied for the surrounding ambient pressures of − 0.8 bar and 0.8 bar. Figure 10a,b show the deformation of the acetonitrile LDRA with an air cavity when it is placed in ambient pressure of − 0.8 bar and 0.8 bar, respectively. By filling up the air cavity with Ecoflex forming a LDRA without any air cavity, the corresponding deformed shapes are also shown in Fig. 10c,d for the two similar pressures. All of the displacements are shown in the same scale at all points on the surfaces. With reference to Fig. 10a,b, for the LDRA with an air cavity, obvious changes in displacement are observed in the horizontal and vertical directions. At the normal ambient pressure (0 bar), external pressure exerting on the surfaces of the LDRA (with air cavity) is balanced out by the internal air pressure inside the air cavity. When there is a reduction of ambient pressure (< 0 bar), the internal air pressure becomes higher than the surrounding. Since air tends to exert pressure in all directions of the cavity due to its free moving molecules, it pushes the interior wall of the Ecoflex and expands the air cavity. This process inflates the air cavity and causes the liquid dielectric to bend outward until the pressure has achieved equilibrium with the atmospheric pressure. Increase in the air cavity’s volume causes the resonant frequency to increase, as can be observed in Fig. 9b, due to the decrease in the effective dielectric constant of the antenna.

For high ambient pressure (> 0 bar), on the other hand, the internal air pressure is lower than the surrounding ambient pressure, causing the whole structure to get compressed. In this case, the resonant frequency decreases. Without having an air cavity inside the LDRA, as can be seen in Fig. 10c,d, the antenna structures are unable to deform when the external pressure is applied. The overall structure remains unchanged due to the near incompressibility of both Ecoflex and acetonitrile. It is obvious that the existence of the air cavity makes the pressure sensitivity of the structure much higher. The importance of incorporating air cavity has also been explained in our previous research^12^.

## Conclusion

For the first time, a dual-functional annular LDRA has been proposed for sensing ambient pressure. Organic liquid dielectric with high permittivity and low loss has been successfully employed for designing a compact LDRA. A soft, flexible, and deformable elastomer platform has been fabricated using soft-lithography technique for holding the liquid dielectric. The central air cavity of the annular DRA has been proven to be crucial so that the antenna structure is sensitive to changes in the ambient pressure. Simulations and experiments have successfully demonstrated the functionality of the design idea proposed by this project. It has been shown that the antenna gain of the proposed acetonitrile LDRA is at least 2 dB higher than that of the water LDRA in the frequency range of 1.8–2.8 GHz. The air pressure sensing feature of the proposed LDRA is sensitive enough (270 MHz/bar) to detect any changes in the ambient pressure. The proposed LDRA can be applied for detecting air pressure in sensitive environments such as airplane cabin and pressure chamber, and the data can be transferred through the LDRA in wireless mode.

## Supplementary information

Supplementary Information 1.
