# Supplementary material for: Compact organic liquid dielectric resonator antenna for air pressure sensing using soft material
Source: Sci Rep. 2020 Sep 10;10:14907. doi: 10.1038/s41598-020-72021-7 (PMC7483734; doi:10.1038/s41598-020-72021-7)
Supplement: Supplementary file 1 — Supplementary Information 1. [file 41598_2020_72021_MOESM1_ESM.pdf]

Compact Organic Liquid Dielectric Resonator Antenna for Air Pressure Sensing Using Soft Material

Jen-Hahn Low[1], *Pei-Song Chee*[2,*], *Eng-Hock Lim*[1], *and Kim-Yee Lee*[1]

[1]Department of Electrical and Electronic Engineering, Universiti Tunku Abdul Rahman, Kajang, 43000, Malaysia

[2]Department of Mechatronics and Biomedical Engineering, Universiti Tunku Abdul Rahman, Kajang, 43000, Malaysia

[*]cheeps@utar.edu.my

1. Calculation of pressure sensitivities

The pressure sensitivities of the sensor at different tilting angles can be calculated from the gradient (m) of the curves in Fig. 9(b). They can therefore be obtained from the curve-fitted equation [$y(\text{GHz}) = mx(\text{bar}) + c$], as shown in Table S1:

Table S1. Pressure sensitivities of the sensor at different tilting angles

Tilting Angle	Equation	Sensitivity
0°	$y = -0.27009x + 2.4$	270 MHz/bar
5°	$y = -0.26397x + 2.4$	264 MHz/bar
10°	$y = -0.25215x + 2.4$	252 MHz/bar
15°	$y = -0.23775x + 2.4$	238 MHz/bar
20°	$y = -0.2158x + 2.4$	216 MHz/bar
25°	$y = -0.19344x + 2.4$	193 MHz/bar
30°	$y = -0.15575x + 2.4$	156 MHz/bar
40°	$y = -0.09273x + 2.4$	93 MHz/bar
50°	$y = -0.04691x + 2.4$	47 MHz/bar
60°	$y = -0.01084x + 2.4$	11 MHz/bar
70°	$y = -0.0x + 2.4$	0 MHz/bar
